# Shotgun Label-free Proteomic Analysis of Clubroot (*Plasmodiophora brassicae*) Resistance Conferred by the Gene *Rcr1* in *Brassica rapa*

**DOI:** 10.3389/fpls.2016.01013

**Published:** 2016-07-11

**Authors:** Tao Song, Mingguang Chu, Rachid Lahlali, Fengqun Yu, Gary Peng

**Affiliations:** ^1^Department of Agriculture and Agri-Food Canada, Saskatoon Research and Development CenterSaskatoon, SK, Canada; ^2^Canadian Light Source Inc.Saskatoon, SK, Canada

**Keywords:** shot-gun proteomics, gene ontology, global proteome machine, canola, clubroot

## Abstract

Clubroot, caused by the plasmodiophorid pathogen *Plasmodiophora brassicae*, is one of the most serious diseases on Brassica crops worldwide and a major threat to canola production in western Canada. Host resistance is the key strategy for clubroot management on canola. Several clubroot resistance (CR) genes have been identified, but the mechanisms associated with these CR genes are poorly understood. In the current study, a label-free shotgun proteomic approach was used to profile and compare the proteomes of *Brassica rapa* carrying and not carrying the CR gene *Rcr1* in response to *P. brassicae* infection. A total of 527 differentially accumulated proteins (DAPs) were identified between the resistant (with *Rcr1*) and susceptible (without *Rcr1*) samples, and functional annotation of these DAPs indicates that the perception of *P. brassicae* and activation of defense responses are triggered via an unique signaling pathway distinct from common modes of recognition receptors reported with many other plant–pathogen interactions; this pathway appears to act in a calcium-independent manner through a not-well-defined cascade of mitogen-activated protein kinases and may require the ubiquitin-26S proteasome found to be related to abiotic stresses, especially the cold-stress tolerance in other studies. Both up-regulation of defense-related and down-regulation of pathogenicity-related metabolism was observed in plants carrying *Rcr1*, and these functions may all contribute to the CR mediated by *Rcr1*. These results, combined with those of transcriptomic analysis reported earlier, improved our understanding of molecular mechanisms associated with *Rcr1* and CR at large, and identified candidate metabolites or pathways related to specific resistance mechanisms. Deploying CR genes with different modes of action may help improve the durability of CR.

## Introduction

The soil-borne plasmodiophorid pathogen *Plasmodiophora brassicae* Woronin causes the clubroot disease on Brassica crops, one of the most serious diseases on cruciferous vegetables and canola/oilseed rape worldwide (Dixon, 2009). In western Canada where over 8 M ha of canola (*Brassica napus* L) are grown annually, clubroot disease was first found on canola in 2003 (Tewari et al., 2005) and has since become a serious threat to canola production due to severe damage caused on many farms in the province of Alberta and to the rapid spread of disease since its discovery (Gossen et al., 2015). The pathogen can survive in the soil as resting spores for up to 20 years (Wallenhammar, 1996). As a result, many conventional disease management methods are ineffective against clubroot, including cultural practices and fungicide treatments (Wallenhammar, 1996; Tsusihima et al., 2010; Hwang et al., 2014; Peng et al., 2014a). Genetic resistance is generally regarded as the most effective and practical approach to clubroot management, especially on canola (Peng et al., 2014a).

Several clubroot resistance (CR) genes, mostly from European fodder turnips (*Brassica rapa* L. ssp. *rapifera*), have been introduced into Brassica crops including oilseed rape (*B. napus*), rutabaga (*B. napus* ssp. *napobrassica*), and Chinese cabbage (*B. rapa* ssp. *pekinensis*; Yoshikawa, 1981; Bradshaw et al., 1997; Hirai, 2006; Diederichsen et al., 2009; Piao et al., 2009). Canola cultivars carrying a single dominant CR gene have also been released in Canada, but the sources and action modes of these CR genes remain unknown (Rahman et al., 2014). The resistance conferred by a single gene, however, is generally not durable. In fact, the breakdown of CR has been reported on Chinese cabbage, oilseed rape, and canola (Matsumoto et al., 2012; Diederichsen et al., 2014; Strelkov et al., 2015). To improve the durability of CR, CR genes with different modes of action may be employed by pyramiding or rotation and a better understanding of resistance mechanisms associated with specific CR genes can lay a good foundation for this approach.

Eight loci of CR genes have been reported previously from *B. rapa*, including *Crr1, Crr2, Crr3, Crr4, CRa, CRb, CRc*, and *CRk* (Matsumoto et al., 1998; Suwabe et al., 2003; Hirai et al., 2004; Piao et al., 2004; Sakamoto et al., 2008), although the resistance mechanisms are unclear for any of these CR genes. A few studies looked at the molecular mechanisms of clubroot pathogenesis using susceptible *Arabidopsis thaliana* ecotypes and found altered host metabolism due to *P. brassicae* infection, including induced carbohydrate and flavonoid metabolism (Evans and Scholes, 1995; Siemens et al., 2006; Päsold et al., 2010). In addition, the phytohormones cytokinin and auxin were also related to clubroot development (Evans and Scholes, 1995; Devos et al., 2006). The Arabidopsis ecotype Bur-0 is partially resistant to clubroot through reducing or delaying pathogen-triggered host metabolic diversion and cell enlargement or proliferation (Jubault et al., 2013). Noticeably, the Arabidopsis ecotype Tsu-0 carries the dominant CR gene *RPB1* and is able to prevent typical root swelling despite *P. brassicae* infection (Fuchs and Sacristán, 1996). Similar information from resistant Brassica crop species may help identify unique mechanisms associated with specific CR genes and allow for more judicious employment of the CR genes in crop cultivars for durable CR.

Previously we evaluated 955 Brassica accessions and identified a range of CR candidates from a wide range of sources, including *B. rapa, Brassica nigra*, and *Brassica oleracea* (Peng et al., 2014b). Further studies characterized the CR gene *Rcr1* based on resistance mapping, and transcriptomic analysis based on RNA sequencing identified over 2000 differentially expressed genes (DEGs) in plants carrying *Rcr1* upon *P*. *brassicae* infection (Chu et al., 2013, 2014). Functional annotation of these DEGs showed that several defense-related biological processes, including signaling and metabolism of jasmonate and ethylene as well as defensive deposition of callose, were up-regulated substantially in plants carrying *Rcr1* (Chu et al., 2014). In contrast, several DEGs involved in the metabolism contributing to clubroot symptom development, such as auxin biosynthesis (Devos et al., 2006) and cell growth/development, showed lower transcriptional levels. These results indicated several potential modes of action by *Rcr1* in conferring resistance to clubroot.

In the present study, a proteomic approach was used, following the transcriptomic study of *Rcr1* (Chu et al., 2014), to gain further insights into the regulation of CR by this CR gene at a post transcriptional level. Proteomics has been used in studying plant responses to biotic stresses based on differentially accumulated proteins (DAPs) as well as their functional annotation (Mehta et al., 2008; Quirino et al., 2010). Two-dimensional electrophoresis (2-DE) used to be used commonly to quantify and compare proteins in different samples (Gao et al., 2012; Wu J. et al., 2014; Wu S. et al., 2014) but technical challenges have limited its application in the post-genomics era, especially for large-scale global profiling of proteome. Quantitative proteomics, especially the use of label-free shotgun techniques, has become a popular approach to replace the 2-DE in studying plant–pathogen interactions (Novo et al., 2014). Critical metabolic or signaling pathways may be identified via functional annotation of DAPs, complementing the findings from other studies such as transcriptomic analysis. This is the first report on using the quantitative proteomics in studying mechanism of CR.

## Materials and methods

### Plants, pathogen, and inoculation

The plant/pathogen materials as well as the inoculation protocol have been described in detail previously (Chu et al., 2014). Briefly, the F1 generation derived from a crossing between the clubroot resistant Pak Choy (*B. rapa*) cv. Flower Nabana (FN) and a susceptible *B. rapa* canola line “ACDC” was used throughout the experiment. ACDC is a self-compatible double haploid line, highly susceptible to clubroot (Peng et al., 2014b). Pathotype 3 of *P. brassicae*, the predominant pathogen race found on canola in western Canada (Strelkov et al., 2007), was used throughout the study. A resting-spore suspension of *P. brassicae* (1 × 10^7^ spores/ml) was applied at 5 ml/plant immediately after seeding and inoculated plants were kept in a growth room until sample collection. ACDC served as the susceptible control to ensure successful inoculation.

### Marker-assisted sample collection

Due to the heterozygosity of FN, the F1 generation segregated for resistance (*Rcr1* present) and susceptibility (*Rcr1* absent) with a ratio at about 1:1 (Chu et al., 2014). To separate the resistant/susceptible plants, genomic DNA from the first true leave of each plant was extracted at 7 days post inoculation (dpi) and tested for the presence/absence of *Rcr1* using the polymerase chain reaction (PCR) with the flanking markers MS1-3 (5′-AAAACAAATATCC-ACCACG-3′ and 5′-CTCAATCCCACAACCTG-3′) and A3-28 (5′-GAGGCCTCCTTTTCTG-GTTT-3′ and 5′-CCGGAGAAGTTTGATTCGAG-3′; Chu et al., 2014). Only the plants with consistent genotype designation based on both markers were selected and used for the experiment. The effectiveness of these markers has been validated (Rahman et al., 2014). The whole root system of each plant was removed at 15 dpi, when secondary infection had likely occurred but clubbing symptoms were still absent (Sharma et al., 2011; Deora et al., 2013). The roots were rinsed with tap water to remove attached soil and debris. Three biological replicates were used for each treatment, each consisting of nine root samples bulked to alleviate the variation from individual plants. All samples were snap-frozen in liquid nitrogen and kept at −80⋅C until protein extraction. Additional 10 plants from each of the inoculated genotype groups were kept in the growth room and assessed for clubroot symptoms at 26 and 42 dpi to determine the success of inoculation (Kuginuki et al., 1999). The disease symptoms consistently matched the designation of genotyping based on the markers.

### Protein extraction and profiling

Each replicate of bulked root samples was pulverized to a fine powder in liquid nitrogen with mortar and pestle. About 100 mg of sample were used for protein extraction using a filter-assisted sample preparation method (Wisniewski et al., 2009). Briefly, a sample was suspended in 8 M urea with 50 mM Tris-HCl (pH 7.6) and 3 mM DTT, sonicated for 10 s, and incubated in an Eppendorf thermomixer (Fisher Scientific, Pittsburgh, PA) at 40⋅C and 1000 rpm for 20 min. Samples were then centrifuged and the supernatant was transferred to a 30 k Amicon MWCO device (Milipore, Etobicoke, ON) and centrifuged at 13,000 *g* for 30 min. The deposit in the centrifuge tube was buffer exchanged with 8 M urea containing 100 mM Tris-HCl, and then alkylated with 15 mM iodoacetamide. The urea concentration in the extract was then diluted to 2 M using the Tris-HCl buffer (pH 7.6). Protein samples were digested by trypsin at 1:100 (enzyme to substrate ratio) and 37⋅C in a thermomixer (1000 rpm) overnight. Digested peptides were then collected by centrifugation at 13,000 g for 30 min, and a portion of the digested peptides (~20 mg) was desalted using C18 stop-and-go extraction (STAGE) tips (Rappsilber et al., 2007). For each sample a C18 STAGE tip was activated with methanol, conditioned with 60% acetonitrile, 0.5% acetic acid followed by 2% acetonitrile, and then 0.5% acetic acid. Samples were loaded onto the tips and desalted with 0.5% acetic acid. Peptides were eluted with 60% acetonitrile, 0.5% acetic acid, and lyophilized in a Savant Speed Vac (Thermo Scientific, Wilminton, DE) for ~2 h to near dryness.

All protein samples were analyzed with UHPLC-MS/MS (Thermo Scientific). Liquid chromatography was performed on an Easy-nLC 1000 UHPLC system, with the mobile phase A solution consisting of 97.5% MilliQ water, 2% acetonitrile, and 0.5% acetic acid. Mobile phase B solution was 99.5% acetonitrile and 0.5% acetic acid. The 240-min LC gradient ran from 0% B to 35% B over initial 210 min, and then to 80% B for the remaining 30 min. Samples were loaded directly into the column (50 cm × 75 μm I.D.) packed with 2 m C18 media (Thermo Scientific). The LC was interfaced to a Q-Exactive quadrupole-Orbitrap mass spectrometer via nano-electrospray ionization using an Easy Spray source with an integrated column heater set at 50⋅C. An electrospray voltage of 2.2 kV was applied. The mass spectrometer was programmed to acquire, by data-dependent acquisition, tandem mass spectra from the top 20 ions in the full scan from 400 to 1200 m/z. Dynamic exclusion was set to 15 s, singly-charged ions were excluded, isolation width was set to 1.6 Da, full MS resolution to 70,000 and MS/MS resolution to 17,500. Normalized collision energy was set to 25, automatic gain control to 1e^6^, max fill of MS to 20 ms, max fill MS/MS to 60 ms, and the underfill ratio to 0.1%.

### Protein identification

Mass spectrometry RAW data files were converted to MGF format using the msConvert and processed through the Global Proteome Machine (GPM) software using the X!Tandem CYCLONE 2011.05.01.1 search engine (http://www.thegpm.org; Craig and Beavis, 2003, 2004). The peptide sequences of annotated proteins derived from the Chinese cabbage (*B. rapa* ssp. *pekinesis*) Chiifu-401 reference genome (V1.2; http://brassicadb.org/brad) was manually inputted into GPM as the reference database for the search of tandem mass spectra generated from the LC-MS/MS. Frequently observed “contaminant” peptides were also included in the database by incorporating the common Repository of Adventitious Proteins (cRAP) developed via the GPM organization (ftp://ftp.thegpm.org/fasta/cRAP). Search parameters were set as 20 ppm and ±0.2 Da mass tolerance for precursor and garment ions, respectively, and full tryptic specificity with one possible missed tryptic cleavage would be allowed. Fixed modification was set as carbamidomethylation of cysteine, and variable modification included methionine and tryptophan oxidation/dioxidation, asparagine, and glutamine deamidation. A cut-off expectation value of 0.1 was required for all proteins identified. The false positive rate (FPR) was calculated with GPM and set at < 1% using the preset parameters of the program. In addition, the ρ-score of each search was calculated to determine the model quality, ranging from 0 (completely random match) to 100 (non-random match). For each genotype, only proteins that were identified in all three replicates with total spectral counts of six or more were included in the final data set with the exclusion of reversed database hits and contaminants (Monavarfeshani et al., 2013). The mass spectrometry proteomics data have been deposited to the ProteomeXchange Consortium via the PRIDE partner repository with the dataset identifier PXD004425.

### Identification of differentially accumulated proteins (DAPs)

The abundance of identified proteins was measured on the basis of normalized spectral abundance factors (NSAFs). For the specific protein k in the sample i, for example, the NSAF_k_ was calculated by dividing the total spectral counts (SpC_k_) by the estimated protein length (L_k_) to the sum of all proteins identified in the sample i. A spectral count of 0.5 was added to all spectral counts initially to compensate for null values, allowing the log transformation of the NSAF-values prior to statistical analysis (McDonald, 2009). Average NSAF-values over three biological replicates were used to calculate the fold change between resistant and susceptible treatments, and a *t*-test was performed to identify DAPs between the resistant and susceptible treatment, with a cut-off value at *P* ≥ 0.05. The collection of DAPs was subjected to functional annotation.

### Functional classification

Functional annotation of DAPs was performed using Blast2GO (https://www.blast2go.com; Conesa et al., 2005). The peptide sequences of all DAPs were extracted and submitted to NCBI for BLAST search using the Blast algorithm (http://blast.ncbi.nlm.nih.gov/Blast.cgi) using default parameters with Viridiplantae (taxid: 33,090) as the organism filter. The BLAST results were downloaded as xml files and manually inputted into Blast2GO for gene ontology (GO) mapping. Gene expression patterns of identified DAPs were visualized by the Mapman software (http://mapman.gabipd.org/web/guest; Thimm et al., 2004). The log-base-2 transformed fold changes of DAPs were used in the Mapman analysis. The mapping file of *B. rapa* was generated using the Mercator software and same reference peptide sequences used for the GPM search described earlier.

## Results

### Analysis of proteomics data

**Figure 1:**
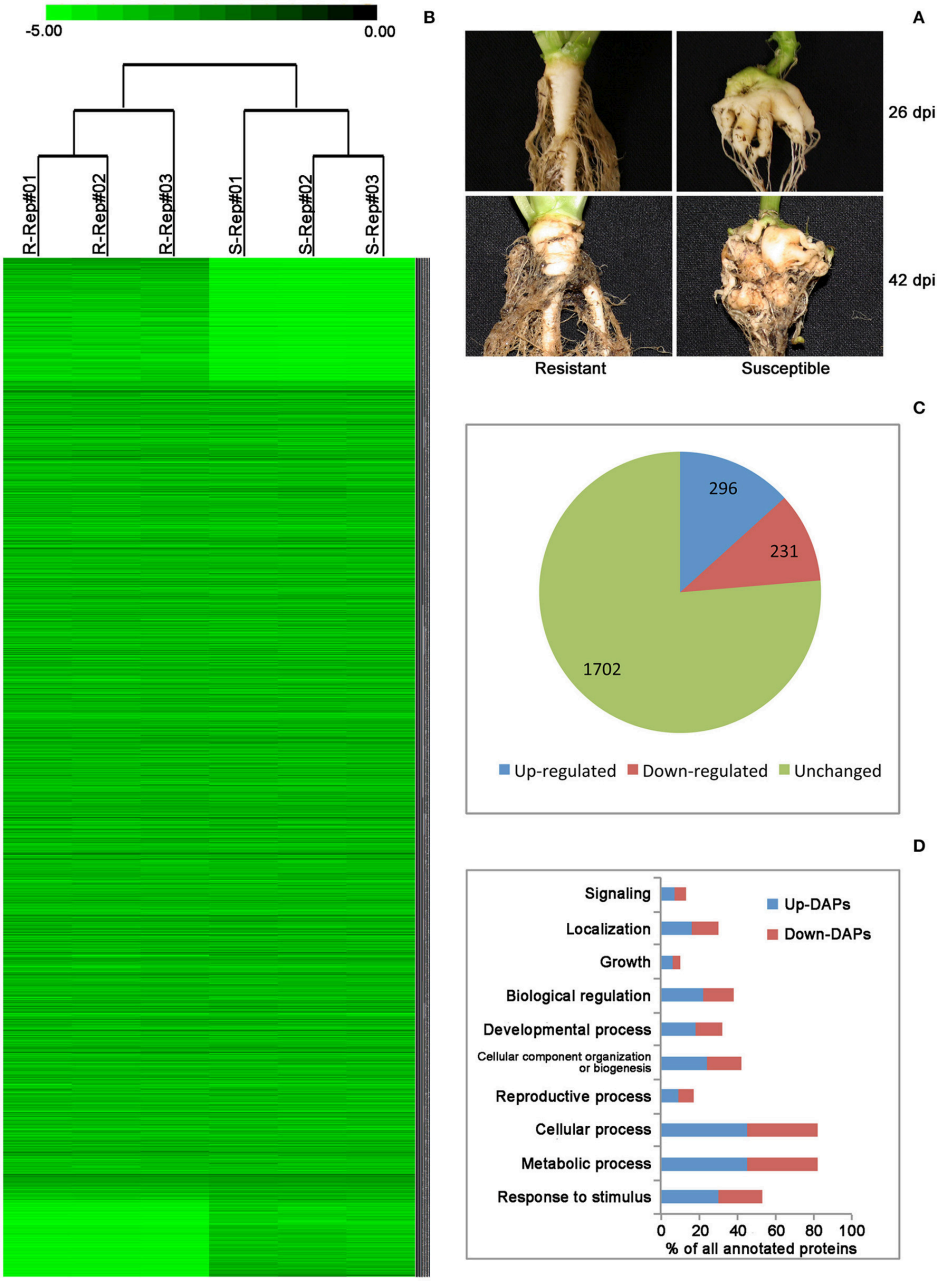
**(A)** Roots of *Brassica rapa* plants carrying *Rcr1* (resistant) and not carrying *Rcr1* (susceptible) at 26 and 42 days post inoculation (dpi) with *Plasmodiophora brassicae*. Club symptoms did not develop on plants carrying the resistance gene *Rcr1*. **(B)** A color map of 2229 non-redundant proteins identified in three biological replicates of resistant and susceptible plants. The darker color represents greater abundance, as determined by normalized spectral abundance factor (NSAF)-values. R, Resistant; S, Susceptible; Rep, Replicates. **(C)** Among the 2229 non-redundant proteins, 527 were differentially accumulated with 296 being up-regulated and 231 down-regulated. **(D)** Functional classification of the differentially accumulated proteins (DAPs).

Successful root infection by *P. brassicae* was confirmed by clubroot symptoms on all plants carrying no *Rcr1* at 26 and 42 dpi, respectively, while none of the plants carrying the CR gene showed any root swelling (Figure 1A). Based on the reference peptide sequences of *B. rapa* Chiifu-401, a total of 2002 and 1859 proteins were identified, respectively, from samples carrying *Rcr1* (resistant—R) and not carrying *Rcr1* (susceptible—S; Table 1), with a total of 2229 non-redundant proteins (including those found in both R and S, as well as exclusively in R or S samples; Table S1, Supporting Information). NSAF-values are attached to show the abundance of protein identified and the low FPR-value found for each replicate, which showed the stringency in adopting the dataset. Cluster analysis, a quality-control measure used for shotgun proteomics based on the correlation between expression profiles among replicates, showed that biological replicates were separated clearly based on the presence or absence of *Rcr1* (Figure 1B). This indicates that the results are highly reproducible.

**Table 1:** **Summary of Proteins Identified by GPM search**.

**Genotypes**	**No. identified proteins**			ρ**-Value**^a^**/FPR**^b^ **(%)**			**No. proteins**
	**Rep#1**	**Rep#2**	**Rep#3**	**Rep#1**	**Rep#2**	**Rep#3**	
Resistant^c^	3297	3302	3602	93/0.71	92/0.70	93/0.70	2002
Susceptible^c^	3228	3187	3153	93/0.71	93/0.67	93/0.70	1859

a *ρ-value is calculated by GPM software to evaluate the quality of the search. The value ranges from 0 (indistinguishable from purely stochastic results) to 100 (putatively all true positives)*.

b *The FPR is the false positive rate*.

c *Plants carrying (resistant) and not carrying (susceptible) the CR gene Rcr1, respectively*.

### Identification and functional annotation of differentially accumulated proteins (DAPs)

The label-free quantitative proteomic analysis characterized the differences in protein synthesis between samples carrying and not carrying *Rcr1* in response to *P. brassicae* infection. Among the total 2229 proteins, the *t*-test (*p* ≤ 0.05) identified 527 DAPs (Table S2, Supporting Information), with 296 DAPs being increased and 231 decreased in resistant samples relative to susceptible ones (Figure 1C). Of the total 527 DAPs, 523 were successfully annotated with GO terms using Blast2GO (Figure S1, Supporting Information) and sorted into major biological Processes (Figure 1D), including signaling, localization, growth, biological regulation, organization of cellular components, or biogenesis, response to stimulus, and reproductive, cellular, developmental and metabolic processes.

### Functional classification of DAPs

**Figure 2:**
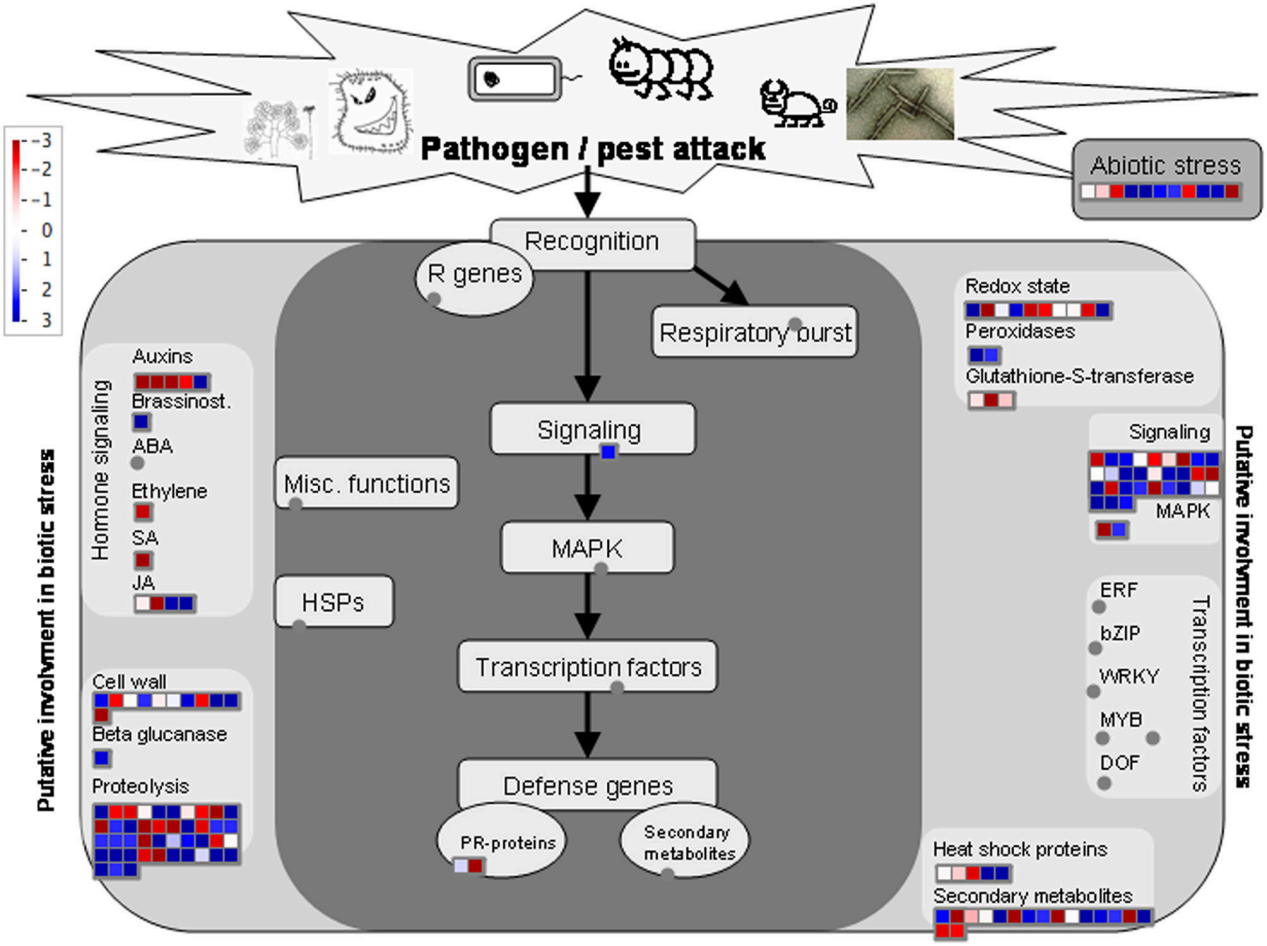
**Annotation of 145 DAPs in relation to signaling pathways involved in biotic/abiotic stress responses based on the analysis using the software Mapman**. The blue and red colors indicate up- and down-regulation, respectively, and gray circles indicate no DAPs identified in these categories. The annotation information is summarized in Table S4 (Supporting Information).

**Figure 3:**
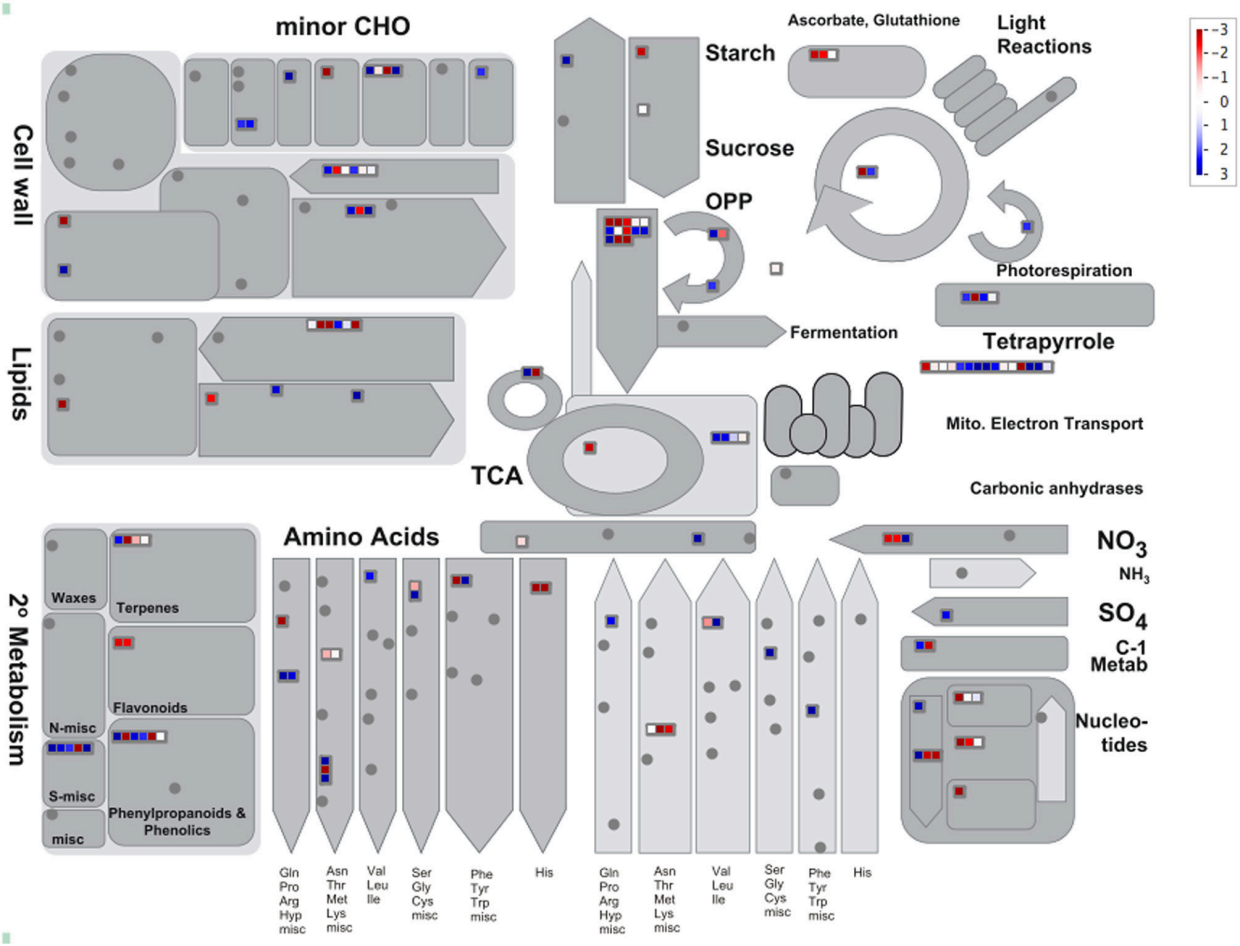
**The visualization of 139 DAPs in metabolism categories using Mapman**. The blue and red colors indicate up- and down-regulation, respectively, and gray circles indicate no DAPs identified in these categories. The annotation information is also summarized in Table S4 (Supporting Information).

The expression pattern of DAP was visualized using the Mapman for insights into the biological context of DAP between resistant and susceptible samples (Table S4, Supporting Information). When mapped to biotic stress, 145 of the DAPs were successfully assigned with a bin code involved in redox regulation, signaling, secondary metabolism, cell wall metabolism, proteolysis process, and response to abiotic stresses (Figure 2). Additionally, 139 of the DAPs were also mapped to several metabolic pathways, including cell wall construction, amino acid metabolism, glycolysis process and mitochondrial electron transport (MET), as well as secondary metabolism including lignin metabolism and sulfur-containing glucosinolate metabolism (Figure 3) catalyzed by the myrosinase. Several primary metabolism processes were also differentially regulated in resistant samples, with MET being enhanced and glycolysis process reduced (Figure 3; Table S4, Supporting Information).

Among the 145 DAPs assigned to the “biotic stress,” 38 were associated with proteolysis in resistant samples, with 23 and 15 showing increased and decreased abundance, respectively(Figure 2; Table S4, Supporting Information). One proteolysis-related DAP (linked to Bra010706) with increased abundance encodes a cysteine proteinase, and 13 other proteolysis-related DAPs were associated with ubiquitin-dependent protein catabolic processes (Table S3, Supporting Information). Many proteins involved in calcium signaling pathways were down-regulated in resistant samples, including one calmodulin (Bra034317), one calcium-dependent protein kinase (Bra013719), and two calcium binding proteins (Bra021896 and Bra016564), as well as a DAP (Bra004784) encoding a protein similar to the mitogen-activated protein kinase 6 (MAPK6) in Arabidopsis. In contrast, the DAP (Bra011137) which encodes a protein similar to MAPKK2 was up-regulated, and so was the one (Bra017049) which encodes a protein similar to E3 ubiquitin-protein ligase HOS1 (At2g39810) found in Arabidopsis (Table S3, Supporting Information). The *B. rapa* EDS1 protein (Bra018085) was also up-regulated. Several DAPs related to reactive oxygen species (ROS) metabolism were observed in resistant samples (Table S3, Supporting Information); the abundances of two protein disulfide isomerase (PDI: Bra010413 and Bra005546) were significantly increased upon infection. Additionally, one cytosolic ascorbate peroxidase, one catalase and two membrane steroid-binding proteins (MSBPs) associated with redox regulation were down-regulated (Table S3, Supporting Information). We also observed that the abundance in Δ^1^-pyyroline-5-carboxylate synthase (Bra005012) was decreased in resistant samples (Table S3, Supporting Information).

## Discussion

As the first attempt of using the quantitative proteomics to decipher CR mechanisms, this study has provided important clues to the modes of action associated with the CR gene *Rcr1* and highlighted the possibility of using this tool to differentiate the mode of action among CR genes. The label-free LC-MS/MS proteomic analysis is efficient for characterizing differences in proteomes between resistant and susceptible samples, with a total of 527 DAPs identified. Surprisingly, only a few of them could be related to the prior transcriptomic analysis (Chu et al., 2014), suggesting some of the post-translational events are able to regulate the protein metabolism without significant changes in transcriptomes. The biological context of these DAPs can be further analyzed by mapping them into different categories based on Mapman ontology, thus linking specific functions to the CR.

### Proteins involved in responses to *Rcr1*-mediated clubroot resistance

Proteolysis and signaling: Among the DAPs assigned to the “biotic stress,” many were associated to proteolysis with either increased or decreased abundances in the resistant samples upon pathogen infection. One of the noteworthy DAPs with increased abundance is linked to the gene Bra010706, which encodes a cysteine proteinase similar to *RD19* in Arabidopsis (At4g39030) where it is required for resistance against a soil-borne disease caused by the bacterial pathogen *Ralstonia solanacearum* (Bernoux et al., 2008). This resistance is mediated by the gene *RRS1-R* which recognizes the type-III effector PopP2 from the bacterium. When PopP2 is present, both *RRS1-R* and *RD19* can be translocated to the cell nucleus where PopP2 interacts with both proteins (Deslandes et al., 2003; Bernoux et al., 2008). Similarly, *Rcr1* was identified as a putative TIR-NBS-LRR type of *R* gene in our earlier work (Chu et al., 2014). Together these results indicate that this recognition mode via *RRS1-R-*PopP2*-RD19* is potentially a candidate for recognition of *P. brassicae* by *Rcr1*. At the same time, the structural differences between *Rcr1* and *RRS1-R* may show that the subsequent signal-transduction processes in inducing defense responses differ; *RRS1-R* is an atypical TIR-NBS-LRR gene with the presence of a C-terminal WRKY DNA binding domain (Deslandes et al., 2002; Eulgem and Somssich, 2007), and can induce downstream gene transcription directly by re-localization to the nucleus when PopP2 is present (Deslandes et al., 2003). None of the *Rcr1* candidate genes, however, carries any DNA binding domain (Chu et al., 2014). Therefore, additional signal transduction would be required to connect the perception of *P. brassicae* to the expression of downstream genes mediated by *Rcr1*.

In addition to cysteine proteinase, 13 other proteolysis-related DAPs were associated with a process catalyzed by ubiquitin-dependent proteins, and this suggests a possible role for an ubiquitin-related proteasome system in the *Rcr1*-mediated CR. These proteins include five 26S proteasome non-ATPase regulatory subunits (Bra040915, Bra028611, Bra013838, Bra028748, and Bra032291), five 20S proteasome subunits (Bra033011, Bra040561, Bra008402, Bra038162, and Bra008308), an ubiquitin carboxyl-terminal hydrolase (Bra009210), the ubiquitin fusion degradation 1 (Bra030307) and an E3 ubiquitin protein ligase (Bra017049). The ubiquitination system plays regulatory roles in many biological processes upon the perception of pathogen, including oxidative burst, hormone/ion signaling, gene transcription, and programmed cell death (Trujillo and Shirasu, 2010). The ubiquitination of receptors and subsequent activation of signaling pathways have received much attention in recent years, including FLS2 in Arabidopsis (Göhre et al., 2008; Gimenez-Ibanez et al., 2009; Lu et al., 2011), XA21 in rice (Wang et al., 2006), and Cf-9 in tomato (González-Lamothe et al., 2006; Yang et al., 2006). It is possible that ubiquitination-mediated signaling pathways serve also a connection between the perception of *P. brassicae* and activation of downstream defense genes.

In the bacterial flg22–FLS2 interaction on Arabidopsis, flg22 is able to activate both the calcium-dependent protein kinase and MAPK cascades that subsequently induce downstream transcription factors (Asai et al., 2002; Boudsocq et al., 2010). In separate studies, MAPK6 could also modulate calcium flux to regulate Arabidopsis root growth under abiotic stresses (Han et al., 2014, 2015). In the current study, many proteins involved in calcium signaling pathways were down-regulated in resistant samples (Results Section Functional classification of DAPs). In contrast, one DAP which encodes an AtMAPKK2-like protein (Bra011137), was significantly up-regulated. MAPKK2 has been shown to regulate MAPK6 and MAPK4 in response to cold and salt stress (Teige et al., 2004). Moreover, MAPK4 is able to induce salicylic acid-dependent but suppress jasmonic acid and ethylene-dependent disease resistance by regulating EDS1 and its interacting partner PAD4 (Brodersen et al., 2006). Our previous transcriptomic analysis of *Rcr1* identified that jasmonic acid/ethylene are the major signaling molecules inducing CR while salicylic acid-mediated signaling pathways are not induced (Chu et al., 2014). Thus, the up-regulation of *B. rapa* EDS1 and absence of a partner *B. rapa* PAD4 protein in resistant samples potentially point to an unique mechanism against *P. brassicae* controlled by a MAPKK2-like kinase and other kinase cascades, inducing jasmonic acid and ethylene-dependent signaling pathways. The enhanced expression of MAPKK2-like kinase also points to possible common host responses to cold and disease stresses. In addition to MAPKK2, another putative regulator against cold stresses was also up-regulated in resistant samples and this DAP (*Bra017049*) encodes a protein similar to the E3 ubiquitin-protein ligase HOS1 (At2g39810) in Arabidopsis. HOS1 is required for the ubiquitination of the transcription factor ICE1 and desensitization of the host to freezing (Dong et al., 2006). Since HOS1 is the key to the specificity of ubiquitin complex (Jackson et al., 2000), it is reasonable to deduce that this DAP (Bra017049) may also target a protein similar to ICE1 in *B. rapa* and induce downstream gene transcription against clubroot, in lieu of cold-tolerance responses.

### Other potential host-defense responses

Upon the recognition of invading pathogen, one of the earliest responses from the host cell is the production of reactive oxygen species (ROS; Torres et al., 2006). This oxidative burst is strictly regulated by a group of enzymes, including ascorbate peroxidase, superoxide dismutase, catalase, and glutathione, to maintain the homeostasis of ROS and restrict ROS-induced damage to the cell (Mittler et al., 2004). The current study identified several DAPs related to ROS metabolism; the abundances of two protein disulfide isomerases (PDI, Bra010413, and Bra005546) were significantly increased in resistant samples upon pathogen infection. PDI belongs to the thioredoxin superfamily with a major function in oxidative folding of polypeptide (Wilkinson and Gilbert, 2004; Ellgaard and Ruddock, 2005). A novel PDI identified from the plant *Oldenlandia affinis* is involved in folding of insecticidal cyclotides (Gruber et al., 2007), and increased PDI has also been observed on wheat in response to infection by the hemibiotrophic fungal pathogen *Mycosphaerella graminicola* (Ray et al., 2003). It is possible that PDI may also function similarly in *B. rapa* and generate anti-microbial proteins against *P. brassicae*. Additionally, a cytosolic ascorbate peroxidase, a catalase, and two MSBPs associated with the redox regulation were down-regulated. The decrease in the expression of ascorbate peroxidase and catalase may contribute to the accumulation of ROS that serves as signaling in inducing downstream defense responses (Kottapalli et al., 2007; Li et al., 2011). The MSBPs may play role in regulating the sterol homeostasis that can be important to root cortical infection by *P. brassicae*; the MSBP 1 (MtMSBP1) in the plant *Medicago truncatula* has been shown to be a determining factor for the symbiosis of *Arbuscular mycorrhiza* in the root cortex (Kuhn et al., 2010). The down-regulation of two MSBPs in *B. rapa* carrying *Rcr1* may disrupt secondary infection of root cortex by *P. brassicae* and consequently hinder the clubroot development.

The role of plant secondary metabolism has been well-studied for disease resistance in many host–pathogen systems. In the current study, two major processes of secondary metabolism were identified; the breakdown of sulfur-containing glucosinolates mediated by myrosinase and lignin metabolism (Figure 3). In Arabidopsis, glucosinolate-myrosinase system has been shown to generate secondary metabolites with anti-microbial activity against a broad spectrum of insects and fungal pathogens (Bednarek et al., 2009; Falk et al., 2014). It is unclear if the glycosinolate-myrosinase would function similarly in *B. rapa* as in Arabidopsis due to sketchy information on glucosinolate metabolism in *B. rapa*. In a metabolomic study, however, several phytoalexins putatively deriving from the glucosinolate metabolism were increased in *B. rapa* roots carrying *Rcr1* upon *P. brassicae* infection (Song and Peng, unpublished data), and this would suggest the possibility for anti-microbial agents via the glucosinolate-myrosinase metabolism. Induced cell-wall lignification has been characterized as a host resistant response in many pathosystems, including wheat-*Blumeria graminis* f.sp. *tritici* (Bhuiyan et al., 2009). Lignin can strengthen the physical barrier to infection (Ride, 1983). In the present study, several enzymes involved in lignin biosynthesis were up-regulated in resistant samples carrying the CR gene *Rcr1*, suggesting a potential role for lignin in CR possibly by reinforcing the cell wall to restrict secondary infection in root epidermal and cortical cells. Two up-regulated peroxidases identified may facilitate the cross-linking of monolignols to form the lignin polymer (Francoz et al., 2015).

### Cross-talks between host responses to abiotic and biotic stresses

Many of the molecular events induced by abiotic stresses may also been found in association with biotic stresses, as shown in the current study that the signaling network of plant cells may be shared by both stress-resistance responses. For example, several signaling molecules reported previously being involved in cold-stress resistance are up-regulated in resistant samples carrying *Rcr1*, and this commonality has been discussed extensively above. Furthermore, the oxidative burst is another critical host response to both abiotic and biotic stresses (Torres and Dangl, 2005). In the current study, 11 proteins with increased abundance are assigned to abiotic stress responses for resistant samples upon *P. brassicae* infection (Figure 2), including chloroplastic and mitochondrial head shock proteins, germin-like protein, MLP-like proteins, methytransferse, and desiccation responsive proteins. The specific functions of these DAPs relating to CR mediated by the CR gene *Rcr1* are still unclear at this point.

### Metabolism

Several processes of primary metabolism were also differentially regulated in CR mediated by *Rcr1*, including enhanced MET and reduced glycolysis (Figure 3; Table S4, Supporting Information). Plant defense is an energy-consuming process resulting from enhanced demand on certain metabolism and cytological activities. Glycolysis and MET are two major pathways for plant respiration which can be stimulated during defense responses to generate ATP and carbon skeletons (Bolton, 2009). It has been demonstrated that glycolysis is contributing to disease resistance in several plant-pathogen interactions, including rice-*Rhizoctonia solan* (Danson et al., 2000; Mutuku and Nose, 2012) and wheat-*Puccinia triticina* (Bolton et al., 2008). In our study, however, the expression of proteins related to glycolysis, especially the major regulator phosphofructokinase (PFK), was reduced while those related to MET were up-regulated in resistant samples. This result suggests that MET serve as the major energy source required for defense responses mediated by *Rcr1* against clubroot. It is also possible that the down-regulation of glycolysis serves as another defense response by inhibiting certain pathogen-induced metabolism (carbohydrate or auxin, for example) favoring excessive cell growth and division. During a compatible interaction between Arabidopsis and *P. brassicae*, an increased carbon flow toward glycolysis was observed (Jubault et al., 2013; Schuller et al., 2013). The pathogen-induced arginine catabolism appeared to be inhibited in resistant samples. In Arabidopsis, proline deriving from the arginine catabolism accumulated substantially in a susceptible ecotype upon *P. brassicae* infection while such an accumulation was not observed in the partially resistant ecotype Bur-0 (Jubault et al., 2008). In the current study, the abundance in Δ^1^-pyyroline-5-carboxylate synthase (Bra005012) was decreased in resistant samples. This enzyme catalyzes the rate-limiting step of proline biosynthesis responding to environmental stresses (Zheng et al., 2014). Even two of the enzymes involved in arginine biosynthesis were up-regulated, the down-regulation of this key enzyme in proline biosynthesis may indicate that the *P. brassicae*-induced arginine catabolism and proline biosynthesis are both disrupted in resistant samples carrying *Rcr1*.

## Conclusions

In the present study, the label-free shotgun proteomic approach was used to analyze the interaction between *P. brassicae* and *B. rapa* carrying *Rcr1* for better understanding of molecular mechanisms associated with this CR gene. The results indicate that the pathogen perception by the host carrying *Rcr1* is potentially through a novel signaling pathway in a calcium-independent manner through an unique MAPK cascade and may require the ubiquitin-26S proteasome which has been linked to cold-stress tolerance. A range of biological processes was also identified in resistant samples where they were either up-regulated for host-defense responses or down-regulated for the metabolism favoring disease development. The former include higher ROS accumulation, breakdown of sulfur-containing glucosinolates and lignin biosynthesis, whereas the latter may include decreased glycolysis and arginine catabolism. To our knowledge, this work is the first proteomic study on CR conferred by a specific CR gene in a Brassica crop species. The study provides further insights into the resistance modes of action for *Rcr1* by comparing the results with those from transcriptomic analysis, and identifies additional candidate signaling/metabolic pathways for further biochemical or genetic studies to verify CR mechanisms. Ultimately, we hope employ multiple CR genes based on their modes of action in canola breeding to develop cultivars with more durable resistance against clubroot.

## Author contributions

GP conceived concept of study and obtained research funding. TS, GP, and FY designed the experiments. TS, MC, and RL performed the experiments. TS analyzed the data. TS and PG developed the manuscript. All authors reviewed, commented, and approved the manuscript for submission.

### Conflict of interest statement

The authors declare that the research was conducted in the absence of any commercial or financial relationships that could be construed as a potential conflict of interest.

## Supplementary material

Table S1 **List of proteins identified in resistant (with ***Rcr1***) and susceptible samples, including sequence length, molecular weights, normalized spectral abundance factor (NSAF)-values (Log10 transformed) of each protein**.

Table S2 **Differentially accumulated proteins, with the significance confirmed using a ***t***-test**.

Table S3 **Annotation of differentially accumulated proteins using Blast2GO**.

Table S4 **Functional classification of differentially accumulated proteins using Mapman**.

Figure S1 **Statistics for the annotation of differentially accumulated proteins by Blast2GO**.
