# Supplementary figures and images for: Shotgun Label-free Proteomic Analysis of Clubroot (Plasmodiophora brassicae) Resistance Conferred by the Gene Rcr1 in Brassica rapa
Source: Front Plant Sci. 2016 Jul 11;7:1013. doi: 10.3389/fpls.2016.01013 (PMC4939851; doi:10.3389/fpls.2016.01013)

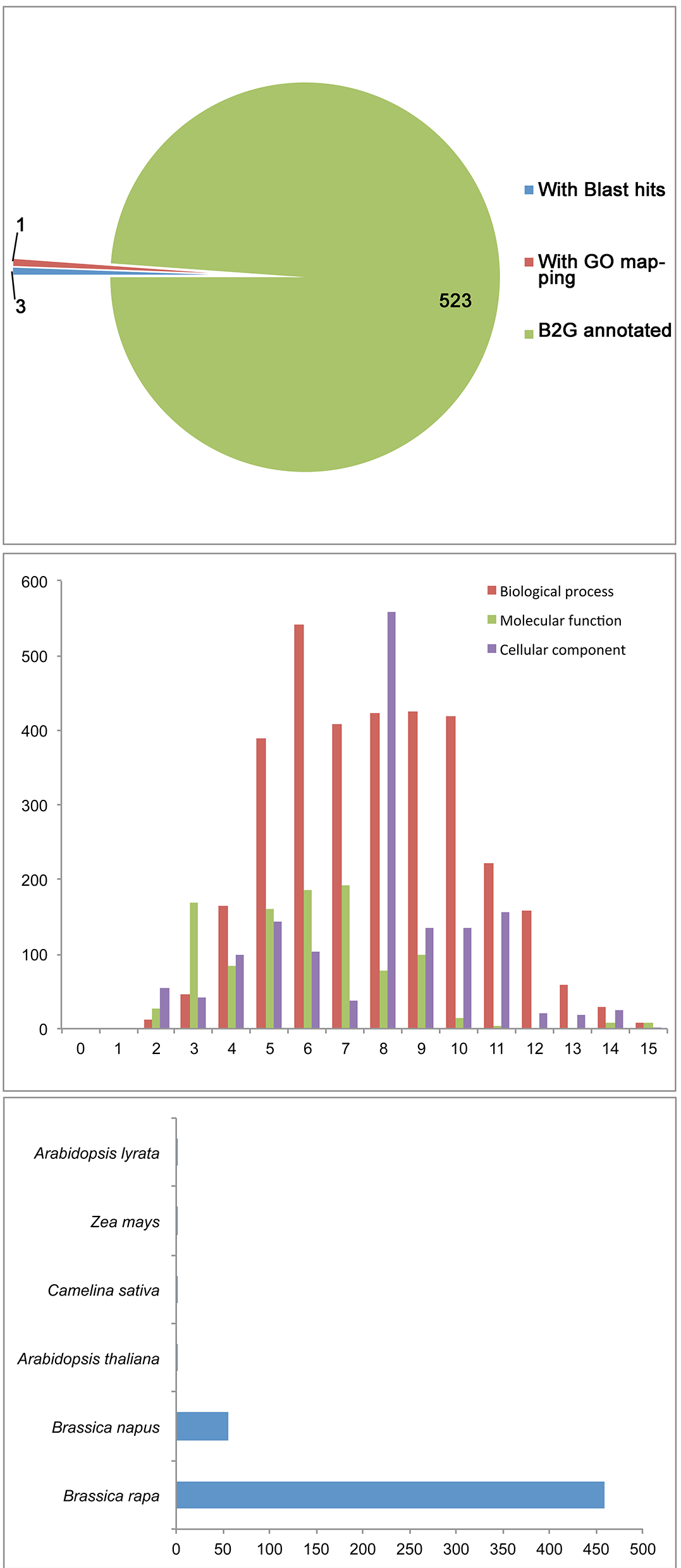

Supplement: Figure S1 — Statistics for the annotation of differentially accumulated proteins by Blast2GO. [file Image1.tif]
